# Injectable Self-Healing Fucoidan-Chitosan Hydrogel Incorporating Polydopamine-Silver-Rutin Nanoparticles for Multifunctional Wound Dressing

**DOI:** 10.3390/molecules31183235

**Published:** 2026-09-13

**Authors:** Peng Ding, Xin Li, Bin Zhang, Ling Wang, Qian Wang, Lei Nie

**Affiliations:** School of Life Science, Xinyang Normal University, Xinyang 464000, China

**Keywords:** fucoidan, chitosan, injectable hydrogel, rutin, PDA, silver nanoparticles

## Abstract

Excessive reactive oxygen species (ROS) and bacterial infection were the important obstacles to impaired wound healing. Herein, by the use of innate bioactivities of natural polysaccharides and flavonoid glycosides, a multifunctional hydrogel was engineered through the Schiff base reaction between oxidized fucoidan (oFu) and lysine-modified chitosan (CS-Ly) for wound dressing. Rutin and silver nanoparticle (AgNP)-modified polydopamine (PDA) nanoparticles were incorporated into the hydrogel matrix, thereby exhibiting favorable ROS scavenging ability and adhesive property. Moreover, the hydrogel demonstrated strong antibacterial activity against *Escherichia coli* (*E. coli*) and *Staphylococcus aureus* (*S. aureus*), with antibacterial rates of 81.8% and 78.2%, respectively. Moreover, the dynamic crosslinking endowed the hydrogel with adjustable degradation behavior and self-healing ability. When cocultured with NIH-3T3 cells, the hydrogel exhibited good biocompatibility. Based on the above results, these multifunctional hydrogels with excellent performance have great appealing potential in clinical materials for wound healing.

## 1. Introduction

Skin wounds caused by external forces and diseases are the most common physical injuries in people’s daily lives. Generally, wound healing is a complex and orderly process of functional recovery and is divided into four overlapped stages: hemostasis, inflammation, proliferation, and remodeling, with the involvement of different cells, including inflammatory cells (e.g., neutrophils and monocytes), platelets and endothelial cells [1,2,3]. If not treated effectively and in a timely manner, it would be at risk of endogenous bacterial infection, resulting in a serious chronic wound and economic burdens [4,5,6]. Therefore, it is necessary to design novel wound dressings to prevent bacterial infection and promote tissue formation. Various biomaterials, such as foams [7,8], films [9,10], nanosheets [11], and hydrogels [12,13,14,15,16], have been developed to accelerate wound closure. Among these materials, hydrogels with excellent properties (e.g., antibacterial activity, self-healing) are widely regarded as promising dressing materials and have attracted extensive attention. This is because hydrogels have a porous structure, possess physicochemical properties similar to the extracellular matrix (ECM), and can act as carriers for drugs or nanoparticles to promote wound healing.

Natural polysaccharides (e.g., chitosan, dextran, and hyaluronic acid) have received extraordinary attention in diverse regenerative medicine and tissue engineering applications owing to their inherent biocompatibility, low immunogenicity, and bioactivity [17,18,19,20,21]. Fucoidan is a marine-derived sulfated polysaccharide extracted from brown seaweeds, characterized by the presence of negatively charged ester-linked sulfate groups that confer a unique structural resemblance to chondroitin sulfate, a natural component of the ECM [22,23,24]. Owing to this distinctive sulfated architecture, fucoidan exhibits a broad spectrum of biological activities, including potent anti-inflammatory, immunomodulatory, and antioxidant properties [25,26]. Notably, its polyanionic nature enables efficient scavenging of ROS and facilitates interactions with endogenous growth factors and extracellular matrix components, thereby modulating cellular behavior in pathological microenvironments [27].

Adhesive antibacterial hydrogels play a critical role in skin wound healing. They not only exhibit strong adhesion at the wound site to prevent detachment during strenuous physical activity but also exert potent antibacterial activity, thereby mitigating infection-induced inflammatory responses and preventing delayed wound healing. Rutin, a natural polyphenolic flavonoid and the principal bioactive constituent of various medicinal plants, exhibits extensive pharmacological properties, including antioxidant [28], anti-inflammatory [29], and antimicrobial activities [30]. However, its clinical translation is significantly hampered by poor aqueous solubility, low bioavailability, and rapid metabolic degradation. Furthermore, its instability in complex physiological microenvironments restricts therapeutic efficacy. To address these limitations, nanotechnology-based delivery systems [31,32], such as polymeric nanoparticles and nanoemulsions, are employed to enhance solubility, protect the compound from degradation, and improve cellular uptake, thereby significantly augmenting its bioavailability and therapeutic potential.

**Scheme 1:**
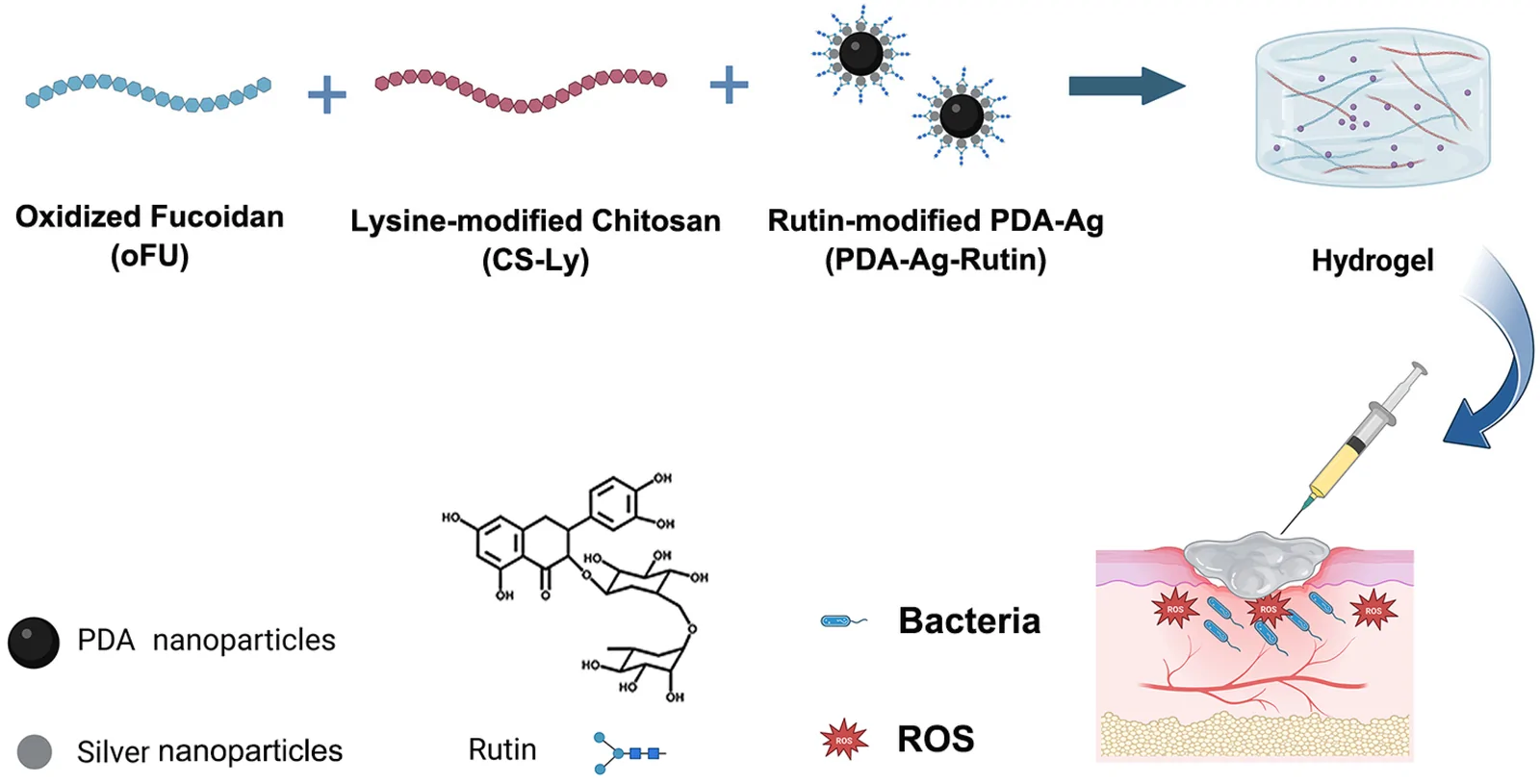
Schematic illustration for the composite hydrogel preparation and its great potential as wound dressings for skin wound management.

Recently, PDA has attracted much attention due to its good biocompatibility and strong adhesion ability to biological tissues [33,34,35]. In this work, the PDA nanoparticles were prepared via oxidative polymerization of dopamine, and their surfaces were subsequently decorated with AgNPs and rutin. Subsequently, as shown in Scheme 1, these PDA nanoparticles were incorporated into a composite hydrogel scaffold consisting of oxidized fucoidan (oFu) and lysine-modified chitosan (CS-Ly). Chitosan was modified with lysine to simulate the glycoprotein components in the ECM and meanwhile enhance its water solubility. Finally, the composite hydrogel exhibited good adhesion, antioxidant, and antibacterial properties, as well as excellent biocompatibility. Additionally, the obtained hydrogel also showed self-healing ability, injectability, and a suitable swelling and degradation rate. Based on these results, we expect the proposed hydrogel to offer new insights into the development of bioactive dressings suitable for wound healing.

## 2. Results and Discussion

### 2.1. Preparation and Characterization of CS-Ly and oFu

**Figure 1:**
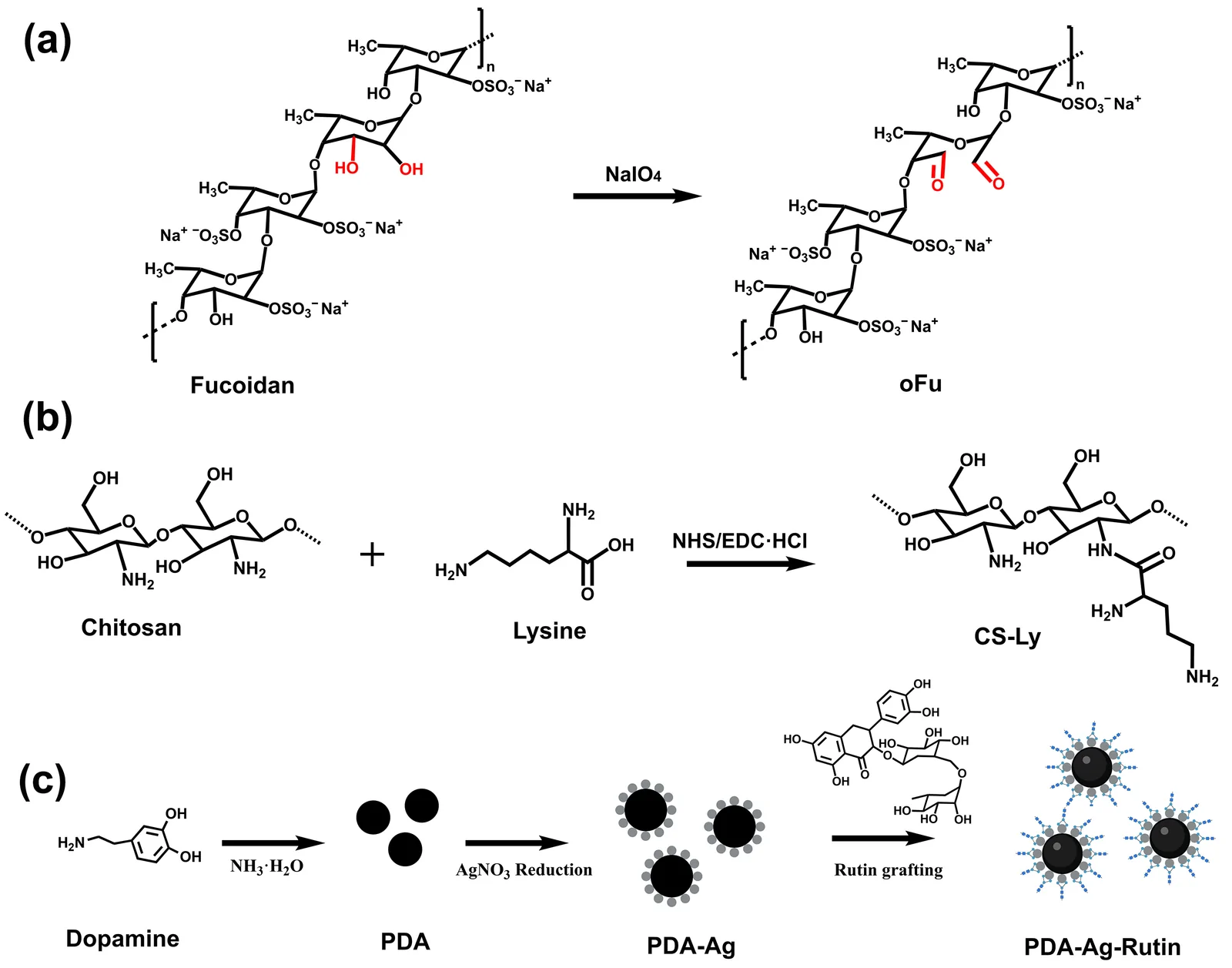
The synthesis route of oFu (**a**), CS-Ly (**b**) and PDA-Ag-Rutin (**c**).

In this work, fucoidan was oxidized to produce oFu with an aldehyde group, which was then reacted with lysine-modified chitosan through a Schiff base reaction to engineer the composite hydrogel. PDA nanoparticles were modified with Ag and rutin to prepare PDA-Ag-Rutin, as presented in Figure 1.

**Figure 2:**
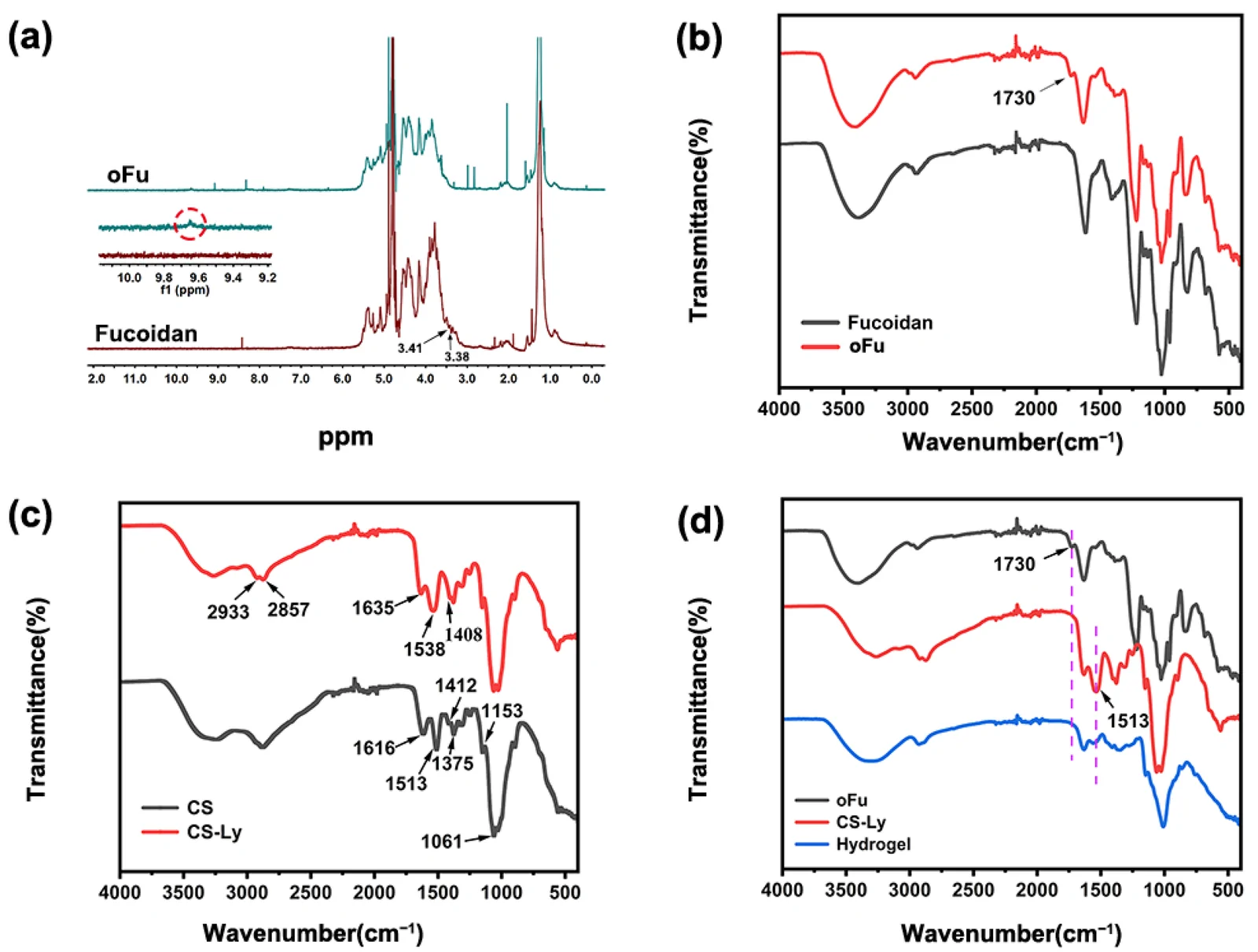
(**a**) ^1^H NMR spectra of fucoidan and oFu. (**b**) FT-IR spectra of fucoidan and oFu. (**c**) FT-IR spectra of CS and CS-Ly. (**d**) FT-IR spectra of oFu, CS-Ly and hydrogel.

The successful preparation of oFu was confirmed by ^1^H NMR and FT-IR spectrum (Figure 2a,b). Compared with fucoidan, the characteristic peak at 9.67 ppm was attributed to the aldehyde group. Moreover, the peak at 3.38 ppm and 3.41 ppm, corresponding to hydroxyl groups in fucoidan, disappeared in the spectrum of oFu after oxidation [22]. In the FT-IR spectrum of oFu, a characteristic peak at 1730 cm^−1^ belonged to the stretching vibration of the –C=O bond from the aldehyde group. These results affirmed the successful preparation of oFu. Moreover, the oxidation degree of oFu was about 28% according to the hydroxylamine hydrochloride method.

In the FT-IR spectra of CS (Figure 2c), the peaks at 1616 cm^−1^, 1513 cm^−1^, 1412 cm^−1^, 1375 cm^−1^ belonged to the –C=O stretching of the amide I band, bending vibration of the N–H (amide II), –C–H bending, and –OH bending, respectively [36,37]. Moreover, the peaks at 1153 cm^−1^ and 1061 cm^−1^ corresponded to the anti-symmetric stretching of the C–O–C bridge and the skeletal vibration of C–O stretching, respectively. Compared with the CS, in the spectrum of CS-Ly, the intensity of –C=O stretching (1635 cm^−1^) decreased and the intensity of –C–H (1408 cm^−1^) bending increased, owing to the formation of an amide bond between lysine and chitosan. Additionally, the peaks at 2933 cm^−1^ and 2857 cm^−1^ were attributed to the anti-symmetric stretching and symmetric stretching of the methylene group from lysine, respectively. These results confirmed the successful grafting of lysine to the chitosan backbone. As shown in Figure S1, compared with CS, the elemental mass percentage of C, N and O elements in CS-Ly increased, substantiating the successful grafting of lysine to CS. Based on the N% of CS-Ly, the degree of substitution of lysine was 21%. Furthermore, the composite hydrogel comprised of oFu and CS-Ly was also characterized by FT-IR. As shown in Figure 2d, there was a stretching vibration peak in the aldehyde group at 1730 cm^−1^, where oFu had a peak but the hydrogel and CS-Ly did not, suggesting that the aldehyde group was consumed by a Schiff base reaction. In addition, compared with CS-Ly, the peak of the hydrogel at 1513 cm^−1^, attributed to the bending vibration of the N–H (amide II), significantly weakened, indicating the occurrence of a Schiff base reaction increased the consumption of the amino groups of chitosan.

### 2.2. Preparation and Characterization of PDA-Ag and PDA-Ag-Rutin

**Figure 3:**
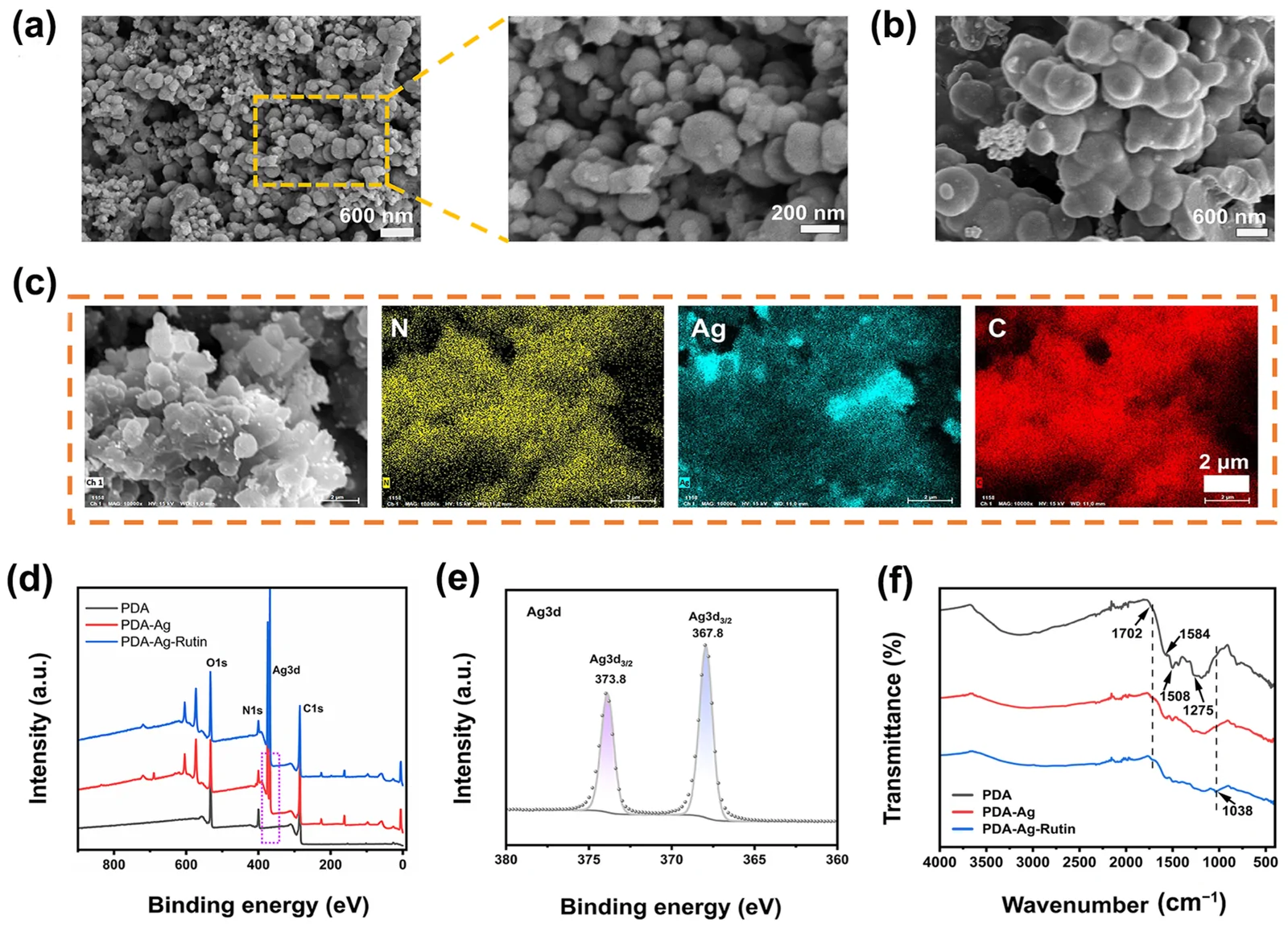
(**a**) SEM images of PDA and (**b**) PDA-Ag. (**c**) EDS elemental mapping (C, N, and Ag) images of PDA-Ag surface. (**d**) XPS survey spectrum of the PDA, PDA-Ag and PDA-Ag-Rutin surface. (**e**) XPS scans of core-level spectra for the Ag element on the PDA-Ag surface. (**f**) The FT-IR spectra of PDA, PDA-Ag and PDA-Ag-Rutin.

As shown in Figure 3a,b, the prepared PDA and PDA-Ag nanoparticles displayed spherical morphology with an average diameter of 288 ± 32 nm and 767 ± 190 nm, respectively. Figure 3c showed the distribution of various elements (C, N, and Ag) on the PDA-Ag surface, which was verified by the characteristic peaks attributed to the corresponding element (C, N, and Ag) in the XPS survey spectra of the PDA-Ag surface (Figure 3d). The high-resolution spectrum of Ag exhibited two characteristic peaks that arose from spin–orbit coupling at the binding energy of 374.8 eV and 368.8 eV, belonging to Ag 3d_3/2_ and Ag 3d_5/2_, respectively (Figure 3e) [38]. The splitting of the Ag 3d doublet of Ag was about 6 eV, suggesting that Ag^+^ was reduced to Ag^0^ by PDA with catechol and nitrogen functional groups [39]. In the FT-IR spectrum of PDA (Figure 3f), the characteristic peaks at 1584 cm^−1^, 1508 cm^−1^ and 1275 cm^−1^ were attributed to the stretching vibrations of the –C=O, C=N and C–N–C bond, respectively, suggesting the successful preparation of PDA [40,41]. Compared with PDA, the intensity of the peak at 1702 cm^−1^, corresponding to the stretching vibration of –C=O, increased, and the new peak at 1038 cm^−1^ belonged to the stretching of –C–O–C, confirming the successful synthesis of PDA-Ag-Rutin.

### 2.3. Physical Characterization of Hydrogels

**Figure 4:**
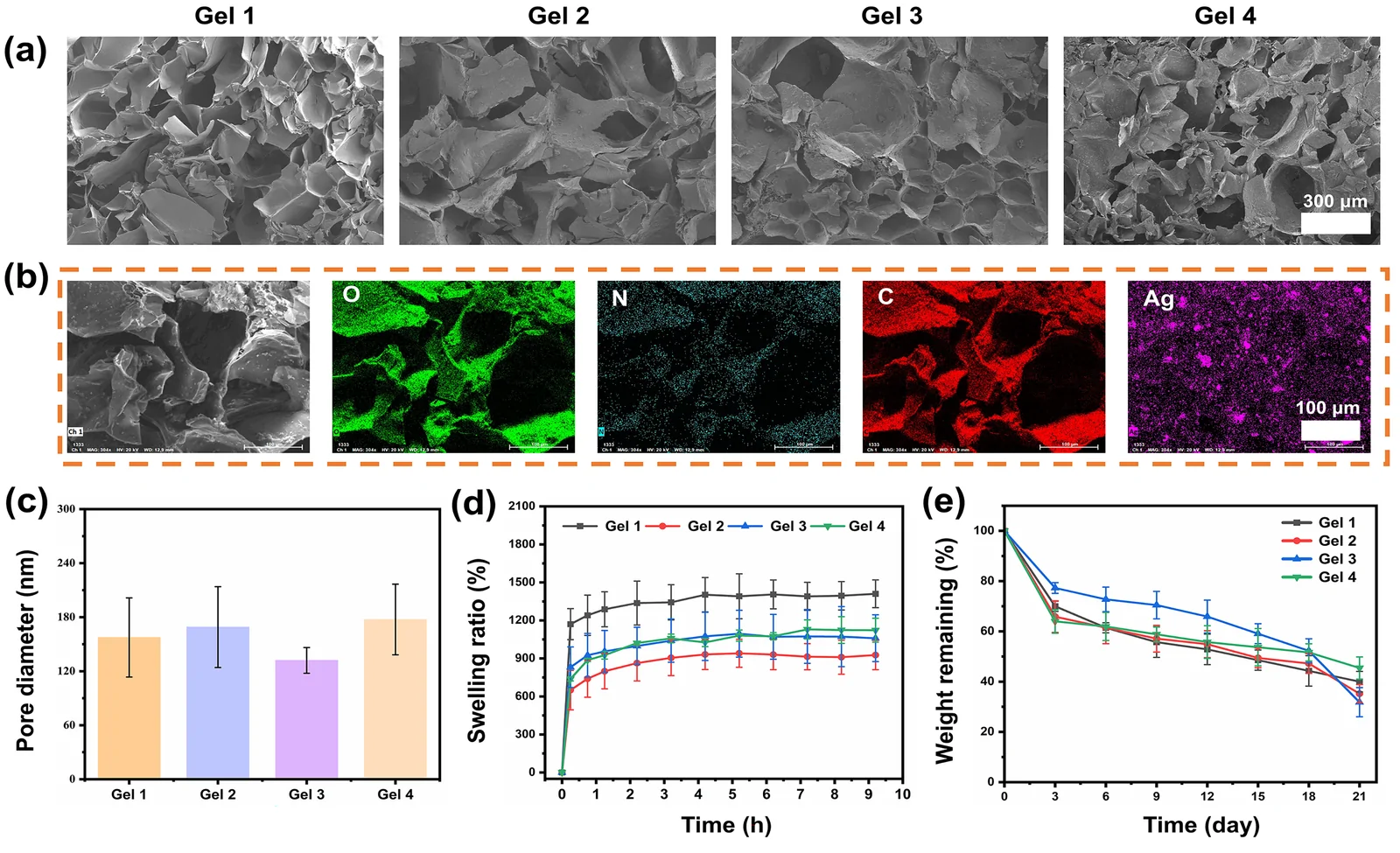
(**a**) SEM images of hydrogels. (**b**) EDS elemental mapping (C, N, O, and Ag) images of Gel 4. (**c**) Pore size distribution, swelling ratios (**d**), and (**e**) degradation curves of hydrogels.

SEM was employed to characterize the internal microstructure of the hydrogels. As shown in Figure 4a, all the prepared hydrogels exhibited an interconnected porous structure, which was highly beneficial for the absorption of skin exudates, nutrient exchange, and cell migration. The EDS analysis of Gel 4 (Figure 4b) further proved that C, N, O and Ag elements were distributed on the Gel 4 surface. In Figure 4c, the pore size for Gel 1, Gel 2, Gel 3 and Gel 4 was 157.5 ± 43.9 μm, 169.1 ± 44.9 μm, 132.2 ± 14.3 μm and 177.5 ± 39.3 μm, respectively. After 9 h of immersion in PBS buffer, the swelling ratios of Gel 1, Gel 2, Gel 3, and Gel 4 were 1410.1%, 926.2%, 1059.7%, and 1122.9%, respectively (Figure 4d). Compared with Gel 1, incorporating PDA nanoparticles might reduce the effective porosity of the hydrogels and then decrease the swelling ratio of the hydrogels.

Furthermore, the degradation profiles of the hydrogels in PBS are presented in Figure 4e. The degradation rates of the prepared hydrogels after 21 days were 61.4%, 64.9%, 68.2%, and 64.4%, respectively. These results demonstrated that the prepared hydrogels possessed tunable degradability. This property is crucial for wound dressings, as it not only eliminates the need for material removal, but also provides essential space for tissue regeneration.

### 2.4. Rheological Properties of Hydrogels

**Figure 5:**
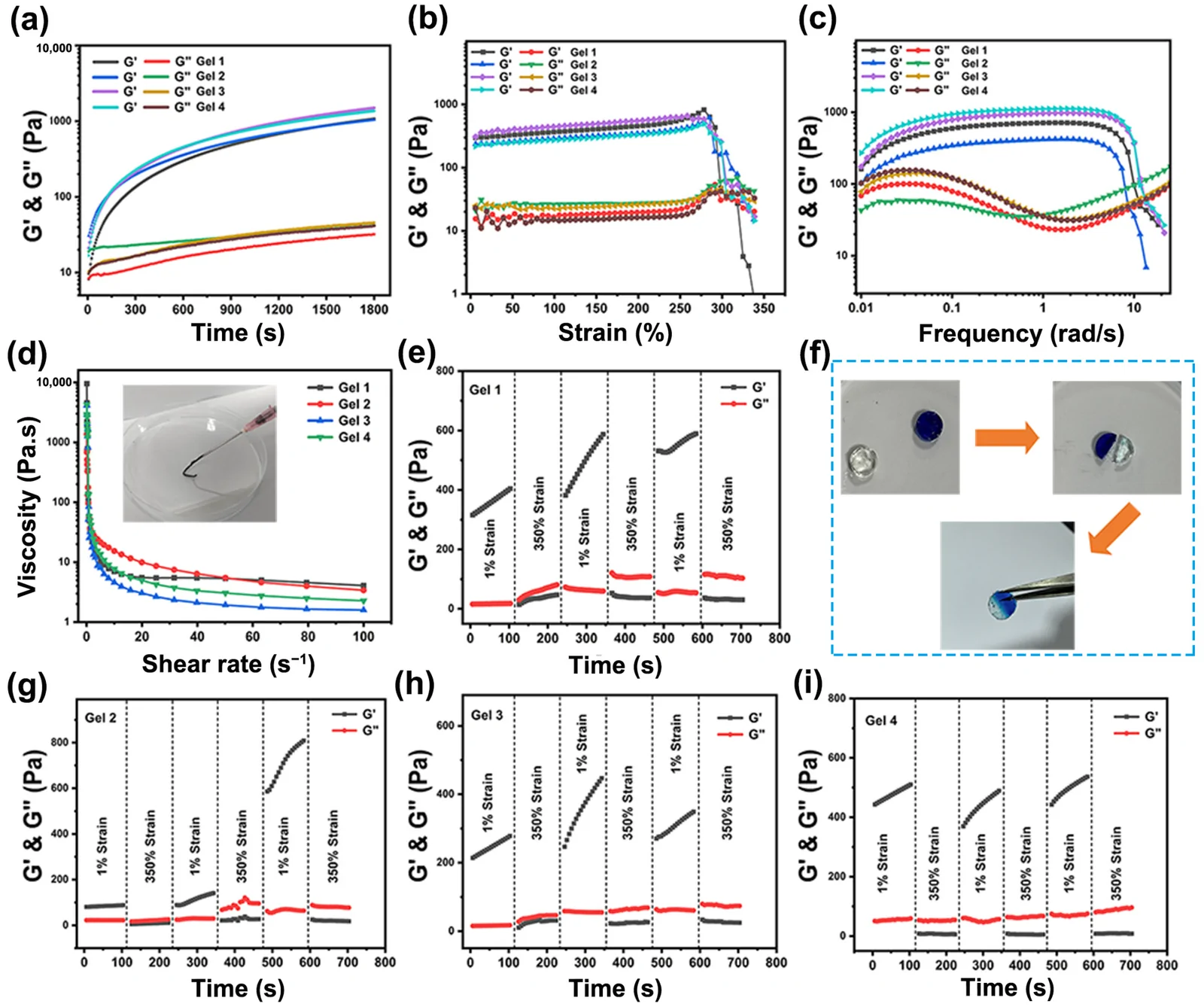
Rheological properties of hydrogels. (**a**) Time sweep, (**b**) strain sweep, (**c**) frequency sweep, and (**d**) viscosity of hydrogels. (**e**) Self-healing of Gel 1 was evaluated by the step strain method. (**f**) Macroscopic self-healing test of Gel 1. (**g**–**i**) Rheological recovery behaviors of Gel 2, Gel 3, and Gel 4.

Hydrogels with tailored mechanical properties are highly desirable for biomedical applications [42]. Therefore, the rheological properties of the prepared hydrogels were thoroughly investigated. As depicted in Figure 5a, during the time sweep test, the storage modulus (G′) of Gel 1, Gel 2, Gel 3, and Gel 4 gradually increased over time and consistently remained higher than the loss modulus (G″), confirming the successful formation of a gel [43]. In the strain sweep test (Figure 5b), G′ remained relatively stable over a broad strain range and was consistently higher than G″, suggesting a robust and stable network structure. When subjected to a higher strain, G′ gradually decreased until a moment where G″ surpassed G′, signifying the collapse of the gel network. Similar results were obtained from the frequency sweep test (Figure 5c).

Furthermore, injectable and self-healing hydrogels not only exhibit excellent conformability to irregular wound surfaces, thereby preventing the leakage of skin wound exudates, but also can autonomously repair themselves upon fracture under external forces, thus preserving structural integrity [44]. As illustrated in Figure 5d, the viscosity of the hydrogels decreased with the increasing shear rate. This shear-thinning behavior facilitated injection through a syringe, as demonstrated in the inset photograph of Gel 2. The self-healing capability was further evaluated via a step strain sweep test (Figure 5e). Taking Gel 1, for example, at a low strain of 1%, G′ was greater than G″, indicating a gel state. At a high strain of 350%, G′ dropped below G″, indicating disruption of the hydrogel network. Remarkably, when the strain was returned to 1%, G′ immediately recovered and exceeded G″, demonstrating the rapid restoration of the hydrogel structure. This self-healing property was also corroborated by a macroscopic self-healing experiment, in which two semicircular hydrogel pieces of different colors seamlessly merged into a single intact circle after 2 h of contact at room temperature (Figure 5f). The observed self-healing and shear-thinning behaviors were attributed to the dynamic Schiff base bonds formed between the unreacted amino groups of chitosan and the aldehyde groups of oxidized fucoidan [45].

### 2.5. Adhesive Strength of Hydrogels

**Figure 6:**
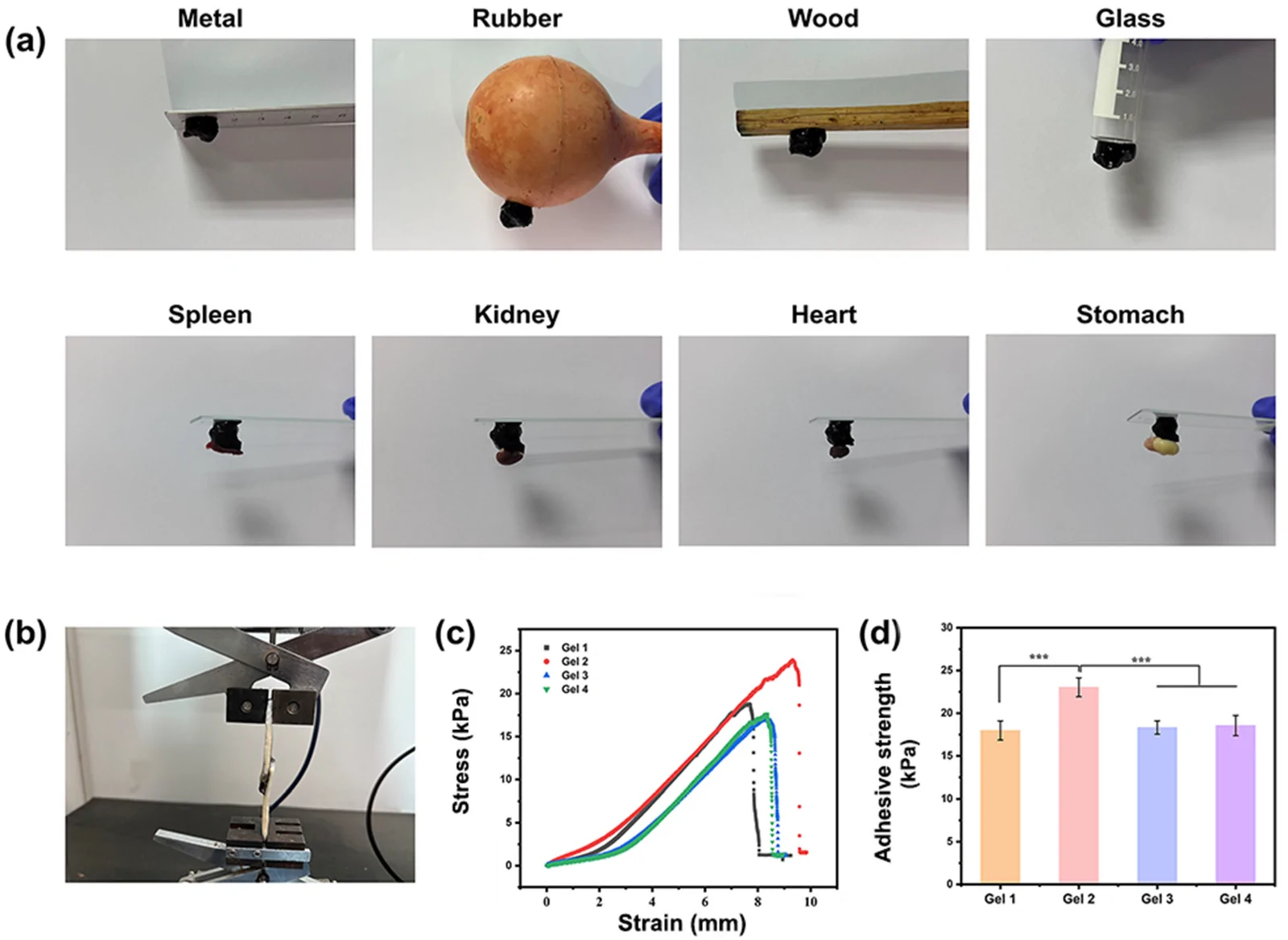
Adhesive property of hydrogels. (**a**) Images of hydrogels adhering to different materials. (**b**) Schematic presentation of the lap shear test and (**c**) Stress–strain curves of the hydrogel. (**d**) Adhesive strength of hydrogels. (*** *p* < 0.001).

Hydrogels with robust adhesiveness can adhere firmly to wounds, preventing detachment during vigorous physical activity and thereby reducing the risk of infection and promoting wound healing [46]. As shown in Figure 6a, the prepared hydrogels demonstrated excellent adhesion to various substrates, including metal, rubber, wood, glass, and different organ tissues. Porcine skin, due to its structural and compositional similarity to human skin, is commonly used as a base substrate to evaluate the adhesive property of hydrogels via a lap shear test. As illustrated in Figure 6b,c, the incorporation of PDA nanoparticles significantly enhanced the adhesiveness of the hydrogels, compared with Gel 1. The adhesive strength of Gel 1, Gel 2, Gel 3 and Gel 4 was 17.9 ± 1.11 kPa, 23.03 ± 1.09 kPa, 18.32 ± 0.78 kPa and 18.55 ± 1.18 kPa, respectively. Notably, Gel 2 exhibited the highest adhesive strength (Figure 6d). This can be attributed to its abundant phenolic hydroxyl groups of PDA nanoparticles, which exhibit strong affinity to the amino or thiol groups of polypeptides and proteins on the skin surface. Moreover, the adhesive strength of Gel 3 and Gel 4 was lower than Gel 2 due to the consumption of catechol groups in reducing Ag^+^ to Ag^0^.

### 2.6. Antibacterial and Antioxidant Properties of Hydrogels

**Figure 7:**
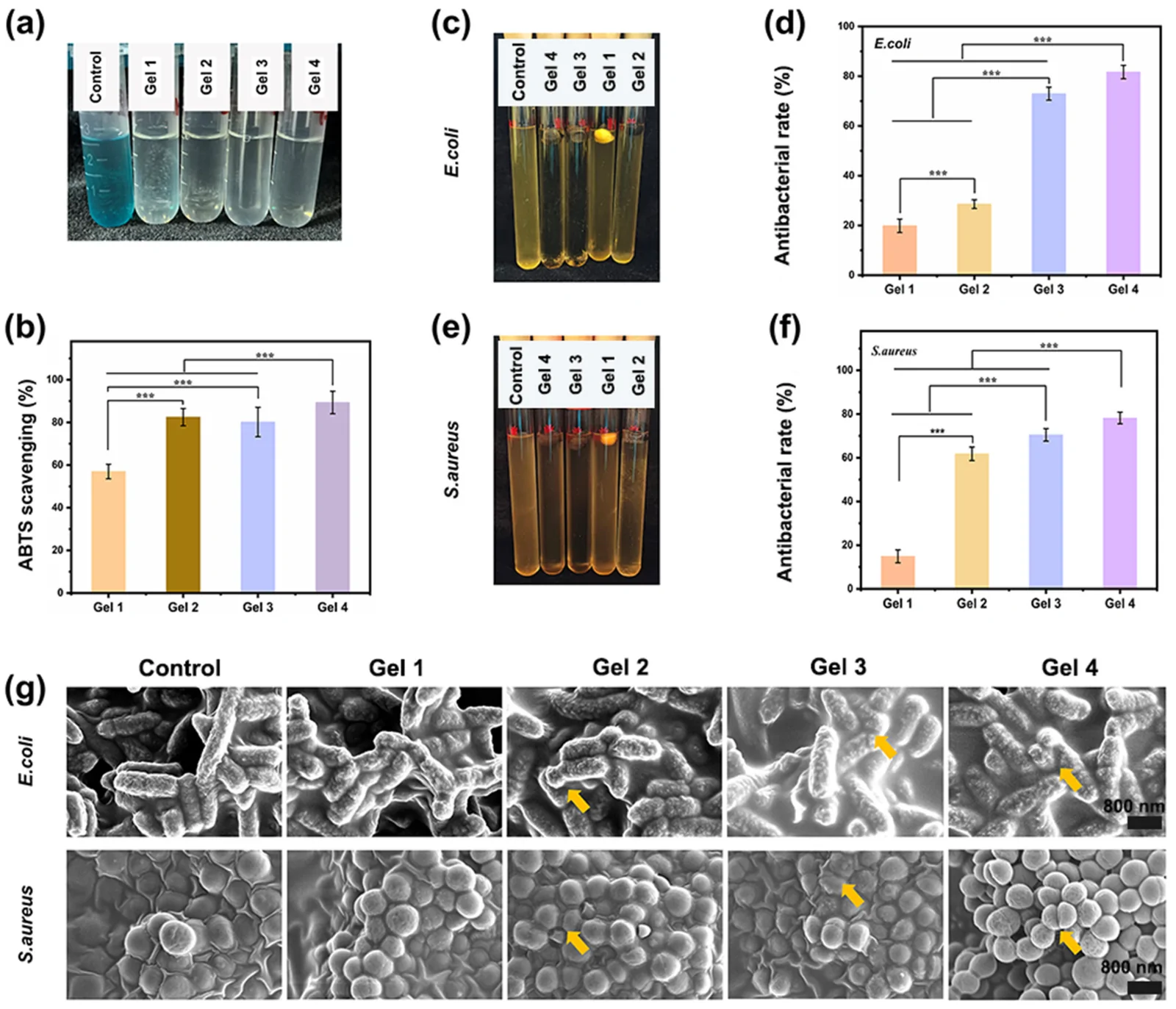
Antioxidant and antibacterial properties of hydrogels. (**a**,**b**) Antioxidant properties of hydrogels. (**c**) Images of the antibacterial effect of hydrogels after coculture with *E. coli* for 6 h. (**d**) Antibacterial rates of hydrogels against *E. coli*. (**e**) Images of the antibacterial effect of hydrogels after coculture with *S. aureus* for 6 h. (**f**) Antibacterial rates of hydrogels against *S. aureus*. (**g**) SEM images of *E. coli* and *S. aureus* after coculture with hydrogels for 6 h. (*** *p* < 0.001).

Skin wounds are highly susceptible to bacterial infection following injury, which often triggers excessive exudate production and abundant ROS generation, thereby impeding the healing process [46]. Therefore, the antibacterial and antioxidant capability of hydrogel dressings are crucial for effective wound management. As shown in Figure 7a,b, the incorporation of PDA nanoparticles, which were rich in phenolic hydroxyl groups, significantly enhanced the antioxidant capacity of the hydrogels, compared with Gel 1. Notably, Gel 4 showed the highest antioxidant efficiency owing to the introduction of rutin with excellent antioxidant activity. Furthermore, the antibacterial efficacy of the hydrogels was also evaluated against *S. aureus* and *E. coli*. As depicted in Figure 7c,d, after 6 h of coculture, the antibacterial rates for Gel 1, Gel 2, Gel 3, and Gel 4 against *E. coli*, were 19.9%, 28.6%, 72.9%, and 81.8%, respectively, while those of the hydrogels for *S. aureus* were 14.9%, 61.8%, 70.5%, and 78.2%, respectively (Figure 7e,f). The SEM images in Figure 7g reveal that, compared with the control group, the bacterial membranes became ruptured or deformable after 6 h of incubation with the hydrogels. These results demonstrated that the introduction of silver nanoparticles and rutin onto the PDA nanoparticles significantly improved the overall antibacterial and antioxidant performance of the prepared hydrogels.

### 2.7. Biocompatibility of Hydrogels

**Figure 8:**
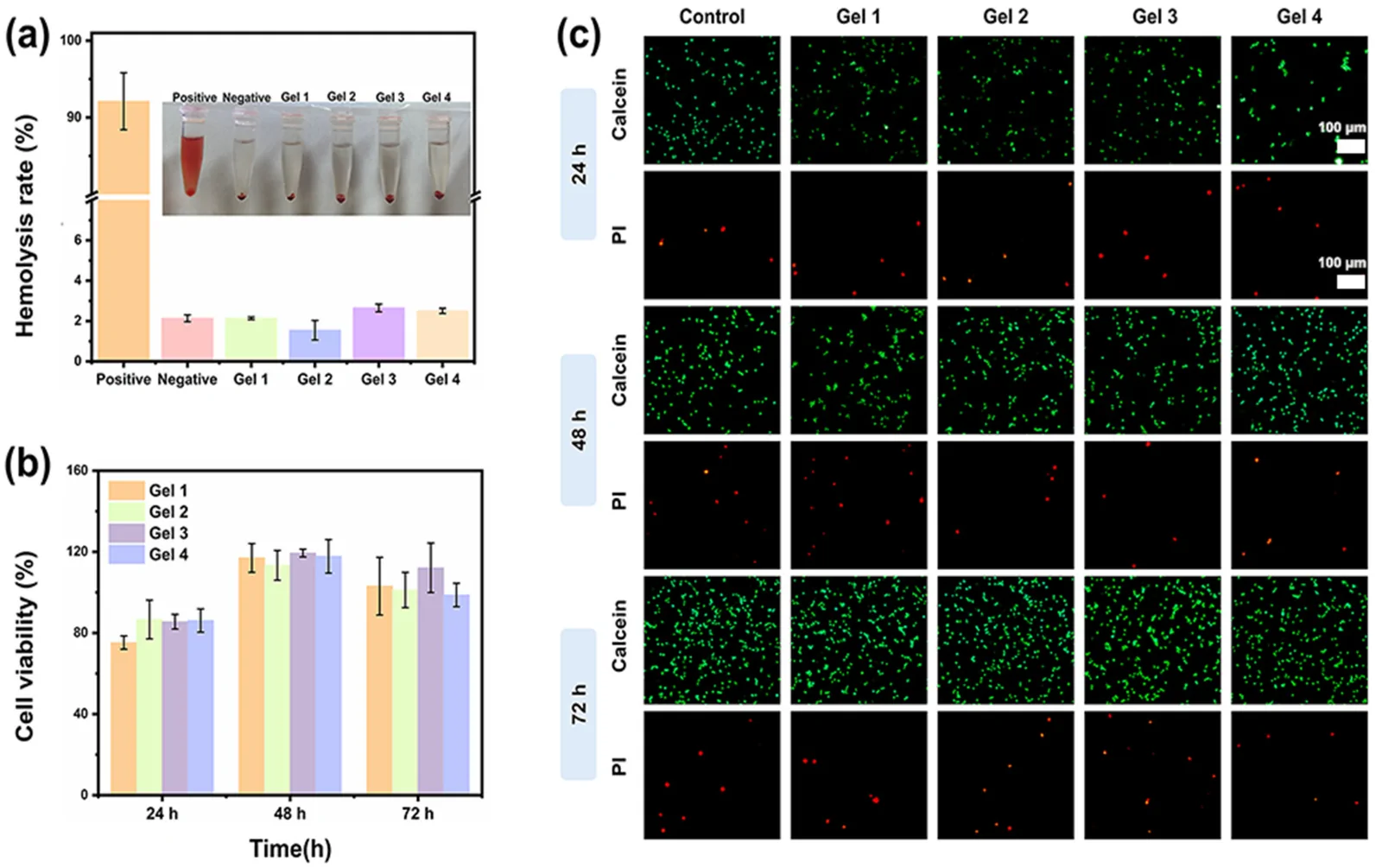
(**a**) Hemolysis test of hydrogels. (**b**) Cell survival rate of NIH-3T3 cells for 24 h, 48 h and 72 h. (**c**) Live/Dead staining of NIH-3T3 cells for 24 h, 48 h, and 72 h.

Biocompatibility and hemocompatibility are indispensable prerequisites for hydrogels used as wound dressings [47]. The in vitro hemolysis assay was employed to evaluate the blood compatibility of the hydrogels. As shown in Figure 8a, the supernatant of the positive groups appeared red, indicating erythrocyte rupture and hemolysis. In contrast, the supernatant from the hydrogel groups remained transparent and colorless, with cells settling at the bottom of the centrifuge tubes. The hemolysis ratios for all samples were below 5%, below the limits set by international standards (ISO 10993-4) [48].

Furthermore, the biocompatibility of the hydrogels was also assessed through coculture with NIH-3T3 cells. As depicted in Figure 8b, after coculturing NIH-3T3 cells with the hydrogels for 1, 2, and 3 days, the cell viability in all the hydrogel groups remained above 75%, satisfying the international biocompatibility test standard for biomedical materials (ISO10993-5) [49]. Consistently, the population of live cells (stained green) in the hydrogel groups increased progressively over time, with no significant dead cells (stained red) (Figure 8c). These results collectively demonstrated the excellent biocompatibility of the prepared hydrogels.

## 3. Materials and Methods

### 3.1. Materials

All chemicals and solvents purchased were of the highest available purity and used as received, unless otherwise stated. Fucoidan (150 kDa) was purchased from Yuanye company (Shanghai, China). Silver nitrate was purchased from Sinopharm chemical reagent Co., Ltd (Shanghai, China). N-hydroxysuccinimide (NHS, 99%), 2-(N-morpholino)ethanesulfonic acid (MES, 99%), rutin (98%), chitosan (with a degree of deacetylation ≥ 95%, molecular weight: 200 kDa), dopamine hydrochloride (98%), sodium periodate (NaIO_4_, 99.5%), 1-ethyl-3-(3-dimethylaminopropyl carbodiimide) hydrochloride (EDC·HCl, 98.5%), L-Lysine (98%), 2,2′-azino-bis(3-ethylbenzothiazoline-6-sulfonic acid) (ABTS, 99%) and potassium persulfate (99.5%) were purchased from Macklin corporation (Shanghai, China).

### 3.2. Synthesis of Lysine-Modified Chitosan (CS-Ly)

Firstly, 2 g of chitosan was dissolved in 200 mL of 1% (*v*/*v*) acetic acid aqueous solution. Then, 1.6 g of lysine, 1.6 g of NHS, and 2.8 g of EDC·HCl were dissolved in 50 mL of MES buffer (pH 5.0, 10 mM) and pre-activated for 30 min. The mixture was then added to the chitosan solution and stirred at room temperature for 24 h. The final pH of the mixed solution was about 6. Finally, the mixed solution was dialyzed using a dialysis membrane (MWCO, 3 kDa) against deionized water for 5 days, and then lyophilized to obtain the CS-Ly.

### 3.3. Synthesis of Oxidized Fucoidan (oFu)

oFu was prepared according to a previous report [22]. Firstly, 3 g of fucoidan was dissolved in 300 mL of deionized water. Then, 6.5 g NaIO_4_ was added to the fucoidan solution and reacted for 24 h away from light. Subsequently, the solution was dialyzed for 5 days and lyophilized to obtain oxidized fucoidan (oFu). The oxidation degree of oFu was determined by the hydroxylamine hydrochloride method [50].

### 3.4. Synthesis of PDA, PDA-Ag and PDA-Ag-Rutin

The PDA nanoparticles were prepared according to a previously reported procedure [51]. Firstly, 2 mL of aqueous ammonia solution (28 wt%) was added to 160 mL 40% (*v*/*v*) ethanol/water solution and stirred for 1 h at room temperature. Then, 0.4 g of dopamine hydrochloride dissolved in 20 mL of deionized water was added to the aforementioned mixed solution and reacted for 24 h in the dark. Finally, acetone was added, and PDA nanoparticles were obtained by centrifugation (8000 rpm, 10 min). The morphology of PDA was characterized using scanning electron microscopy, and particle size was measured with ImageJ (Version, 1.54).

An amount 0.6 g of PDA nanoparticles was added to 300 mL of 10 mg/mL AgNO_3_ solution and stirred at 37 °C for 24 h in the dark. Then, the mixture was centrifuged at 10,000 rpm and washed three times with deionized water to remove unreacted Ag^+^. Subsequently, the nanoparticles were lyophilized to get PDA-Ag. An amount of 0.2 g of rutin was dissolved in 300 mL of ethanol/Tris-HCl (3/7, *v*/*v*) at 40 °C for 2 h. Then, 0.4 g PDA-Ag was added to the rutin solution via ultrasonic oscillation for 2 h. Subsequently, the mixture was reacted for 12 h and then centrifuged at 10,000 rpm for 10 min to get PDA-Ag-Rutin. Finally, the obtained PDA-Ag-Rutin particles were washed three times with anhydrous ethanol and deionized water respectively to remove unreacted Ag^+^ and rutin, and then were subjected to freeze-drying to obtain the final sample.

### 3.5. Preparation of CS-Ly/oFu Hydrogels

Amounts of 0.02 g of PDA, PDA-Ag, and PDA-Ag-Rutin were added to 1 mL of CS-Ly solution (4% *w*/*v*), respectively. Then, the CS-Ly solution was blended with 1 mL of 8% (*w*/*v*) oFu solution in equal volumes to prepare Gel 1, Gel 2, Gel 3, and Gel 4, respectively. Gel 2 indicated that the composite hydrogel only contained 1% PDA, Gel 3 meant that the composite hydrogel only had 1% PDA-Ag, while Gel 4 showed that the composite hydrogel only had 1% PDA-Ag-Rutin. Gel 1 was used as the control group and only contained CS-Ly and oFu.

### 3.6. Fourier-Transform Infrared Spectroscopy (FT-IR)

Fourier-transform infrared spectroscopy (ThermoFisher Nicolet iS50, Waltham, MA, USA) characterization was used to analyze the presence of specific chemical groups of fucoidan, oFu, CS, CS-Ly, PDA, PDA-Ag and PDA-Ag-Rutin. The FT-IR spectra were obtained within the range between 400 and 4000 cm^−1^ with a resolution of 1 cm^−1^, with each spectrum averaged over 64 scans.

### 3.7. Proton Nuclear Magnetic Resonance (^1^H NMR) Spectroscopy

Initially, fucoidan and oFu were completely dissolved in 0.6 mL of D_2_O, respectively. Subsequently, the ^1^H NMR spectra were performed on a 600 MHz NMR spectrometer (JEOL ECZ600R/S3, Tokyo, Japan) equipped with a 14.09 T superconducting magnet.

### 3.8. Morphology of Hydrogels

The freeze-dried hydrogels were immersed in liquid nitrogen for 2 min and then fractured with a blade to obtain uniform blocks. These fragments were then coated with a platinum layer. The morphological structure of the composite hydrogels was analyzed via SEM (Hitachi S-4800, Tokyo, Japan). Additionally, the pore size of the hydrogel samples was measured using ImageJ (1.54).

### 3.9. Swelling and Degradation Rates of Hydrogels

The freeze-dried hydrogels were immersed in 50 mL phosphate-buffered saline (PBS, pH 7.4, 10 mM) at room temperature. Then, the hydrogel was collected from the solution at each predetermined time interval, and excess fluid was removed with the filter paper. The swelling rates of the hydrogels were calculated through the following formula:

(1) $$\mathrm{Swelling}\;\mathrm{rate}\;(\%)=\frac{W_{t}-W_{c}}{W_{c}}\times 100\%$$

where *W_c_* and *W_t_* were the weight of the freeze-dried hydrogel and the weight of the wet hydrogel, respectively.

In vitro degradation test: The freeze-dried hydrogels were immersed in PBS buffer and shaken at 120 rpm. At predetermined time points, the hydrogel samples were retrieved, lyophilized, and weighed. The degradation rate was subsequently calculated using the following formula:

(2) $$\mathrm{The}\;\mathrm{degradation}\;\mathrm{rate}\;(\%)=\frac{W_{i}-W_{s}}{W_{i}}\times 100\%$$

where *W_i_* and *W_s_* represented the weight of the initial and final hydrogel, respectively.

### 3.10. Rheological Properties of Hydrogels

The rheological properties of the hydrogels were determined by a TA rheometer (DHR-2, New Castle, DE, USA) equipped with a parallel plate geometry (20 mm in diameter) at room temperature with a gap of 1000 nm [52]. The time sweep test for 1800 s was conducted by keeping the strain and frequency constant at 1% and 1 Hz, respectively. The strain sweep test was implemented in a strain range of 0.1–350% at a frequency of 1 Hz. The frequency sweep test in the range of 0.1–100 rad/s was carried out at 1% strain. The viscosity of the hydrogels against a shear rate in the range of 0.1–100 s^−1^ was measured. The step strain measurement was carried out by changing the strain (from 1% to 350% with a 100 s interval) for three cycles to evaluate the self-healing behaviors of the hydrogels.

### 3.11. Macroscopic Self-Healing Test of Hydrogels

A bulk hydrogel sample was sectioned into two equal halves. One-half was stained with a methylene blue solution, while the other remained unstained. The two distinct pieces were then brought into intimate contact and allowed to heal at the interface. After 2 h, the self-repairing process was photographed and documented.

### 3.12. Antioxidant Activity

ABTS stable radical solution was prepared according to the previous report [53]. Briefly, 7 mM of ABTS aqueous solution was mixed with 2.45 mM of potassium persulfate solution at room temperature in the dark for 12 h. Under dark conditions, 0.02 g of the hydrogel sample was added to 5 mL of the ABTS working solution, and shaken at 37 °C with a shaking speed of 150 rpm for 30 min. Then, the absorbance value was measured at 734 nm using a microplate reader (SpectraMax 190, Molecular Devices, San Jose, CA, USA). The antioxidant activity was calculated according to the following formula:

(3) $$\mathrm{ABTS}\;\mathrm{Scavenging}\;\mathrm{rate}\;(\%)=\frac{A_{c}-A_{h}}{A_{c}}\times 100\%$$

where *A_c_* and *A_h_* were the absorbance of the control group and the hydrogel group, respectively.

### 3.13. Antibacterial Test

*Escherichia coli* (*E. coli*, ATCC 25922) and *Staphylococcus aureus* (*S. aureus*, ATCC 6538) were used to evaluate the antibacterial activity of the hydrogels according to the previously reported procedure [54]. Briefly, 0.02 g of freeze-dried hydrogel was added into a glass tube with 10 mL of Luria–Bertani (LB) broth and 10 μL of the bacterial suspension (10^6^ CFU/mL). The mixed solution was cocultured at 37 °C for 6 h. The control group contained 10 mL of LB broth and 10 μL of the bacterial suspension (10^6^ CFU/mL) without hydrogels. The optical density of the bacterial suspension was measured at 600 nm using a UV–Vis spectrophotometer (PERSEE T6, Beijing, China). The antibacterial rates of the hydrogels were calculated through the following formula:

(4) $$\mathrm{Antibacterial}\;\mathrm{rate}\;(\%)=\frac{A_{c}-A_{s}}{A_{c}}\times 100\%$$

where *A_c_* and *A_s_* represented the absorbance of the control group and the sample group, respectively.

Moreover, the morphology of the bacteria after coculture with the hydrogels for 6 h was observed using SEM.

### 3.14. Adhesion Performance Evaluation

First, the adhesion performance of the hydrogel on various material surfaces, including rubber, metal, glass, and wood, was evaluated. Subsequently, its adhesion capacity to multiple organ tissues (heart, spleen, stomach, kidney) from Kunming mice (Male, 20–30 g) was investigated. All adhesive interfaces were documented through imaging. Then, the adhesive capability of the composite hydrogels was qualitatively assessed by a lap shear test using fresh porcine skin as the base material [48]. Briefly, porcine skin was purchased from the local supermarket and then excised into rectangular strips measuring 50 mm (length) × 20 mm (width). The subcutaneous fat layer was carefully removed, and the tissue was rinsed three times with anhydrous ethanol before being immersed in PBS for subsequent use. Subsequently, 600 μL of the prepared hydrogel was sandwiched between two porcine skin samples. After 5 min, the mechanical testing was performed using a universal testing system (CMT1000, Sansi Taijie, Zhuhai, China) equipped with a 100 N load cell at a crosshead speed of 2 mm/min. The porcine skin after the experiment was handed over to a certified professional disposal company (qualified for medical/hazardous waste treatment) for centralized, harmless treatment. The entire transfer process was documented via a waste transfer manifest system to ensure full traceability.

### 3.15. Hemocompatibility Evaluation

Fresh blood was obtained from mice (Kunming mice, male, 20–30 g) and collected into EDTA-containing anticoagulant tubes. Red blood cells (RBC) were harvested by centrifugation (3000 rpm, 10 min) and washed with an appropriate amount of 0.9% physiological saline. All animals were handled in strict accordance with the Laboratory Animal Care and Use Guidelines, and approved by the Animal Ethics Committee of Xinyang Normal University, and the ethical approval number is XFEC-2026-074. Hydrogel samples (2.5 mg) were added to 1.5 mL centrifuge tubes containing 1 mL of 0.9% physiological saline and incubated at 37 °C for 30 min. Subsequently, 20 μL of the RBCs suspension was added to the tubes, and the mixture was incubated at 37 °C for an additional 1 h. The absorbance of the supernatant was measured at 540 nm using a microplate reader (SpectraMax 190, Molecular Devices, USA). The positive group consisted of 1 mL of deionized water mixed with 20 μL of RBC suspension, while the negative group consisted of 1 mL 0.9% physiological saline mixed with 20 μL of RBC suspension. The hemolysis ratio was calculated using the following formula [55]:

(5) $$\mathrm{Hemolysis}\;\mathrm{Ratio}\;(\%)=\frac{A_{h}-A_{n}}{A_{p}-A_{n}}\times 100\%$$

where *A_h_*, *A_n_*, and *A_p_* represented the absorbance of the hydrogel group, the negative group, and the positive group, respectively.

### 3.16. Cytotoxicity Evaluation

The cytotoxicity of the hydrogels was evaluated using the Cell Counting Kit-8 (CCK-8, Kumamoto, Japan) assay through the coculture with NIH-3T3 cells (CRL-1658TM, ATCC, Manassas, VA, USA) according to the previous reports [56,57]. Briefly, the hydrogel extracts were prepared according to the ISO 10993-12 protocol [58]. An amount of 100 mg of the hydrogel sample was sterilized by being exposed to ultraviolet light for 2 h and then immersed in 1 mL of medium (Dulbeco’s modified Eagle’s medium supplemented with 10% fetal bovine serum and 1% penicillin–streptomycin) and incubated at 37 °C for 24 h. Subsequently, 1.0 × 10^4^ cells/mL NIH-3T3 cells were seeded into 96-well plates and cultured to reach the logarithmic growth phase. Then, 200 μL of hydrogel extract was added into the wells and the cells were cocultured with the hydrogel extract at 37 °C in a 5% CO_2_ environment for 1, 2, and 3 days, respectively. Then, 10 µL of CCK-8 solution was added to each well, and the absorbance was measured at 450 nm using a microplate reader (SpectraMax 190, Molecular Devices, USA). The experimental setup comprised three groups: the experimental group contained cells, medium, CCK-8 solution, and hydrogel extract, the control group contained cells, medium, and CCK-8 solution, but no hydrogel extract, and the blank group contained medium and CCK-8 solution, but no cells or hydrogel extract. Cell viability was calculated using the following formula:

(6) $$\mathrm{Cell}\;\mathrm{Viability}=\frac{A_{e}-A_{b}}{A_{0}-A_{b}}\times 100\%$$

where *A_e_*, *A_0_*, and *A*_b_ represented the absorbance of the experimental group, control group, and blank group, respectively.

Moreover, after coculture with hydrogel for 24 h, 48 h and 72 h, the morphology of the cells was observed with a fluorescence microscope (Leica TCS SP5 II, Wetzlar, Germany) through Live/Dead staining to evaluate the cytocompatibility of the hydrogels [59].

### 3.17. Statistical Analysis

All experiments were performed in triplicate. All experimental data are expressed as mean ± standard deviation and analyzed by one-way analysis of variance (ANOVA) using SPSS software (Version 27) to investigate statistical difference; a *p* value < 0.05 was considered as significant (* *p*< 0.05, ** *p* < 0.01 and *** *p* < 0.001).

## 4. Conclusions

In conclusion, an injectable and self-healing composite hydrogel was successfully fabricated via a Schiff base reaction between oxidized fucoidan and lysine-modified chitosan. Moreover, rutin and AgNP-modified PDA nanoparticles were encapsulated into the hydrogel matrix. Therefore, the composite hydrogel not only exhibited strong antibacterial efficiency due to the synergistic effects from rutin and AgNPs and achieved an 82% inhibition rate against *E. coli* and a 78% inhibition rate against *S. aureus*, but also showed remarkable antioxidant activity and tissue adhesiveness. Furthermore, the prepared hydrogel demonstrated superior biocompatibility, suitable mechanical properties and biodegradability. Therefore, these findings suggest that the developed hydrogel holds significant potential for clinical applications in advanced wound management.

## Data Availability

The original contributions presented in this study are included in the article/Supplementary Material. Further inquiries can be directed to the corresponding author(s).

## Supplementary Materials

The following supporting information can be downloaded at: https://www.mdpi.com/article/10.3390/molecules31183235/s1. Figure S1. The XPS spectrum of CS (a) and CS-Ly (b).
