# Supplementary material for: Injectable Self-Healing Fucoidan-Chitosan Hydrogel Incorporating Polydopamine-Silver-Rutin Nanoparticles for Multifunctional Wound Dressing
Source: Molecules. 2026 Sep 13;31(18):3235. doi: 10.3390/molecules31183235 (PMC13609611; doi:10.3390/molecules31183235)
Supplement: Supplementary file 1 [file molecules-31-03235-s001.zip › molecules-4558243-supplementary.pdf]

Supporting Information

Injectable self-healing fucoidan-chitosan hydrogel incorporating polydopamine-silver-rutin nanoparticles for multifunctional wound dressing

Peng Ding, Xin Li, Bin Zhang, Ling Wang, Qian Wang and Lei Nie *

School of Life Science, Xinyang Normal University, Xinyang, 464000, China
* Correspondence: nieleifu@yahoo.com; nielei@xynu.edu.cn

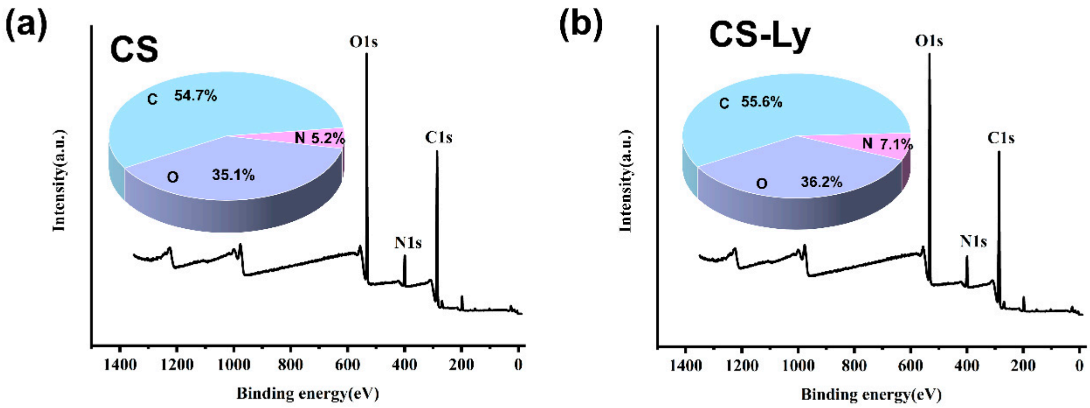

Figure S1. The XPS spectrum of CS (a) and CS-Ly (b).
